# BLENDED GAMING COVID-19 TRAINING SYSTEM (BGCTS) FOR RESIDENTIAL CARE HOMES: A CLUSTER RANDOMIZED CONTROLLED TRIAL

**DOI:** 10.1093/geroni/igad104.1850

**Published:** 2023-12-21

**Authors:** Angela Y M Leung, Terence Lau, Doris Y P Leung

**Affiliations:** Hong Kong Polytechnic University, Hong Kong, Hong Kong; The Hong Kong Polytechnic University, Hong Kong, Hong Kong; The Hong Kong Polytechnic University, Hong Kong, Hong Kong

## Abstract

**Objective:**

This study aims to assess the effect of the Blended Gaming COVID-19 Training System (BGCTS) on infection control practices, compliance rates and knowledge of standard precautions among all staff in residential care homes (RCHs). Design: A 2-arm single-blinded cluster randomized controlled trial. Setting: Thirty RCHs were recruited and randomized. Due to COVID-19 pandemic and there were infected cases in the homes, 17 RCHs refused or delayed the on-site observations. Participants: 212 staff (from 13 RCHs) were randomized to the intervention group (IG) or the control group (CG) according to their RCHs. Intervention: This is a blended mode of training with contents derived from WHO and local guidelines.

**Results:**

After using the BGCTS , the use of gloves and personal protective equipment (PPE) was significantly improved in IG than that of CG (F=43.206, p< 0.001). The IG has significantly higher scores in self-reported infection control practices (SICP) than that of CG (β= 2.619, Wald χ2 95% CI [4.291, 9.426]).

**Discussion:**

BGCTS improved RCH staff’s performance in infection control practices. BGCTS was shown to be an effective training and it is worth to consider for adopting it as routine training for all staff in residential care homes.

